# Quantification of fatty acids in seed oil and important bioactive compounds in Iranian *Rosa canina* L. ecotypes for potential cosmetic and medicinal uses

**DOI:** 10.1038/s41598-023-50135-y

**Published:** 2023-12-20

**Authors:** Ziba Bakhtiar, Ghasem Eghlima, Mehrnaz Hatami, Mohammad Hossein Mirjalili

**Affiliations:** 1 Department of Agriculture, Medicinal Plants and Drugs Research Institute, Shahid Beheshti University, Tehran, 1983969411 Iran; 2 Department of Medicinal Plants, Faculty of Agriculture and Natural Resources, Arak University, Arak, Iran

**Keywords:** Ecology, Evolution

## Abstract

*Rosa canina* L. (Rosaceae), commonly known as the rose hip, is originated from Europe, Africa, and Asia with a long history in medicinal applications. This study aimed to analyze the morphological traits, fatty acids profile, and content of phenolic compounds, anthocyanins, vitamin C, total carotenoid, total phenol, total flavonoid, and antioxidant activity of the fruits of eleven Iranian *R. canina* ecotypes (RCEs). The highest coefficient of variation was obtained in 1000 seed weight (46.57%). The seed oil varied from 8.08 ± 0.17% to 16.91 ± 0.35%. Linoleic (35.41 ± 0.78% to 49.59 ± 0.96%) and eicosanoic (17.67 ± 0.06% to 25.36 ± 0.54%) acids were the predominant fatty acids in the studied samples. The anthocyanin content in the fruits was ranged from 0.98 ± 0.03 to 4.41 ± 0.04 mg cyanidin 3-glucoside/100 g of dry weight (mg C3G/100 g DW). The high content of vitamin C (103.51 ± 1.24–419.70 ± 3.12 mg/100 g DW), total carotenoid (111.22 ± 0.78–206.98 ± 1.25 mg β-carotene equivalents per g of dry weight (mg β-CARE/g DW)), total phenol (52.87 ± 0.82–104.52 ± 0.23 mg GAE/g DW), and total flavonoid (14.20 ± 0.12–25.18 ± 0.47 mg RE/g DW) were observed in the studied samples. Catechin (20.42 ± 0.47–19.22 ± 0.13 µg/g DW) was the major phenolic compound. The high antioxidant activity in the fruits of the plant was recorded in the studied RCEs (IC_50_ = 12.54 ± 0.18–26.33 ± 0.13 μg/ml). A significant correlation between some phytochemical compounds (dependent variable) and morphological features (independent variable) was found. Based on our findings, the fruit of the studied ecotypes can be used for future breeding programs and drug development.

## Introduction

Nowadays, plants have gained special importance in the process of discovering and developing medications due to their specialized metabolites^1,2^. The development of spectroscopy in the nineteenth century rendered it possible to detect specialized metabolites in plants, which sped up their application in medicine production^3^. The separation, identification, and quantification of biologically active compounds in plants play a fundamental role in their use in the pharmaceutical industries^4^.

The genus *Rosa* belongs to the Rosaceae family and consists of 100–250 species^5^. The rose hip (*Rosa canina* L.) is a permanent and deciduous species whose height is between 2 and 3 m. It has imparipinnate compound leaves with 5–7 toothed leaflets and light pink flowers^6^. Rose hip is resistant to diverse environmental conditions (poor and rocky soils and water scarcity). So, it grows in wide regions of Europe, Northwestern Europe, and Western Asia. *Rosa canica* fruits (RCFs) are rich in polyphenols, e.g., flavonoids, anthocyanins, catechin, procyanidin, phenolic acids, including gallic and ellagic acids, kaempferol, apigenin, and resveratrol^7,8^. It is also an invaluable source of various vitamins, especially vitamin C^6^. Its fruits contain high levels of carotenoids, tocopherols, minerals (Ca, K, P, Na, Fe, Mn, and Zn), tannins, organic acids, amino acids, and pectin^9^. Saturated fatty acids (SFA) including palmitic and stearic acid and unsaturated fatty acids (USFA) such as linolenic and linoleic acids were found in the seeds of the rose hip^10^. Lycopene and β-carotene are the most important carotenoids in its fruits^11^.

The RCFs are traditionally used to cure arthritis, rheumatism, gout, sciatica, the cold, and infections, such as influenza, prevent gastritis and stomach ulcer, and treat skin diseases and lesions^12,13^. The most valuable part of the fruit is the pericarp which can be used in various products, such as medicinal products, herbal tea, jam, marmalade, syrup, jelly, and soft drinks, and has recently been employed as an ingredient of probiotic beverages, yogurt, and soup^14–16^. Its seed oil is mainly consumed in the cosmetic and pharmaceutical industries^10,17^.

The morphological traits of the RHFs, such as fruit weight and length, flesh percentage, and thickness are important traits, and their measurement and selection can help develop new cultivars^18^. The morphological and phytochemical diversity that is found in different types of plants is due to the interaction between environmental and genetic conditions. Phytochemical diversity is an important part of yield diversity. Therefore, adequate knowledge of the diversity of yield and economic traits is necessary for assessing genotypes and designing efficient breeding processes to achieve the breeding goals^19^. Research has revealed high phytochemical diversity in different *Rosa* species^11,15,20–22^. The breeding programs of the *Rosa* species have recently focused on the quality and quantity of bioactive compounds, e.g. vitamin C and phenol compounds of fruits^23,24^. Recently, the use of RCFs and their products is increasing^25^. It is important to include wild species that have valuable compounds in breeding programs. The present study analyzed the phenolic compounds, vitamin C content, total carotenoid content (TCC), seed oil yield, and fatty acids in different RCEs in Iran and introduced the best ecotypes for the initiation of breeding programs, cultivation, domestication, and application in pharmaceutical and food industries.

## Materials and methods

### Chemicals

The chemicals used in this study were in analytical grade. Standards and trifluoroacetic acid were supplied from Merck (Darmstadt, Germany). Butylated hydroxytoluene, Folin-Ciocalteu’s reagent, boron trifluoride, hydroxide potassium, sodium hydroxide, sodium nitrite, aluminum chloride, sodium carbonate, metaphosphoric acid, *n*-hexane, acetone, diethyl ether, methanol, and ethanol were purchased from Sigma-Aldrich company (USA).

### Plant material

Fruits of the eleven RCEs were collected from eight Provinces of Iran. The geographical coordinates of the studied areas were shown in Table 1. The fruits were harvested at full ripening time. The distances of 2000 m were considered between the ecotypes in each collection region to avoid sampling clones of the chosen ecotypes. The samples were identified by Prof. Ali Sonboli, and voucher specimens were deposited at the Shahid Beheshti University herbarium (Table 1). The authors confirm that the necessary permissions to collect the samples have been obtained and also the present study complies with the IUCN Policy Statement on Research Involving Species at Risk of Extinction and the Convention on the Trade in Endangered Species of Wild Fauna and Flora.

**Table 1:** Geographical information for the collection regions of *Rosa canina* ecotypes.

No.	Location	Code	Voucher number	Latitude (N)	Longitude (E)	Altitude (m)	MAT (°C)*	MAP (mm)**
1	Alborz-Mohammad shahr	*RC1*	MPH-3204	35° 46′	50° 55′	1320	14.4	247
2	Ardabil-Abibiglu	*RC2*	MPH-3205	38° 16′	48° 32′	1329	9.6	245
3	East Azerbaijan-Ajabshir-Chenar	*RC3*	MPH-3206	37° 58′	46° 03′	1894	16.5	235
4	Kermanshah-Sonqor	*RC4*	MPH-3207	34° 47′	47° 31′	1634	17.1	273
5	Kohgiluyeh va Boyer Ahmad-Gachsaran	*RC5*	MPH-3208	30° 22′	50° 48′	765	23.8	431
6	Markazi-Saveh	*RC6*	MPH-3209	35° 02′	50° 30′	1128	18.2	216
7	West Azerbaijan-Bukan-Borhan	*RC7*	MPH-3210	36° 41′	45° 57′	1647	11.5	335
8	West Azerbaijan-Mahabad-Gavmishan	*RC8*	MPH-3211	36° 23′	45° 44′	1624	13.0	376
9	West Azerbaijan-Mahabad-Maraneh	*RC9*	MPH-3212	36° 32′	45° 34′	1576	12.5	353
10	West Azerbaijan-Sardasht	*RC10*	MPH-3213	36° 27′	45° 54′	1260	14.5	895
11	Zanjan-University of Zanjan	*RC11*	MPH-3214	36° 68′	48° 39′	1577	14.2	200

*MAT, mean annual temperature.

**MAP, mean annual precipitation.

### Morphological analysis

Morphological traits were measured on eleven samples. The data were measured on 30 randomly selected fruit from each ecotype for six quantitative characteristics; fruit length (cm), fruit width (cm), fruit weight (g), pericarp weight (g), 1000 seed weight (g), and seed number per fruit (number). The weight for fruit, pericarp, and 1000 seed was recorded using an electronic balance (0.01 g precision).

### Phytochemical analysis

#### Fatty acid analysis

Crude oil content was determined by the maceration method^26^. Initially, 500 mg dried powdered seed sample was added with 5 ml *n*-hexane and ultrasonicated for 60 min. The mixture was left at 23 ± 2 °C for 72 h and filtered with Whatman filter paper No. 1. *n*-Hexane was evaporated at room temperature. The crude oil was kept in airtight, colored bottles at − 18 °C until further analysis. The plant seed oils were esterified as methyl esters before analysis^27^ and then injected into a gas chromatography mass spectrometry (GC–MS) system (Agilent Technologies, 7890A, USA). The GC–MS system was installed with a universal column (HP5; 30 m, 0.325 mm, 0.25 μm; Agilent J&W GC column). Helium was used as carrier gas at a flow rate of 1.2 ml/min. The column temperature was increased from 150 to 240 °C at 3 °C/min and maintained for 20 min. The samples (1 ml) were injected in the split mode of 1:100. Determination and identification of fatty acids were used in the reference samples (NU-CHEK-PREP company, USA).

#### Extraction of phenolic compounds and HPLC analysis

Phenolic compound extraction was quantified as described by Demir et al.^22^. Concisely, 1 g powdered pericarp was ultrasonicated (Elma, S120H, Germany) with 100 ml methanol/water/ trifluoroacetic acid (90:10:0.02 v/v/v) for 30 min and centrifuged (Centrifuge Rotanta 460r, Hettich, Germany) at 1400*g* for 10 min at 4 °C. The extract was dried in a rotary evaporator (Heidolph Instruments GmbH, Schwabach Germany) at 35 °C. The extract was solved in 1 ml methanol and then filtered (0.22 µm). Phenolic compounds were determined using a high-performance liquid chromatography-photodiode array, with a Waters 2695 separations module equipped with a C_18_ column (250 × 4.6 mm) and a UV detector (Waters 2487). Mobile phases were methanol/water/trifluoroacetic acid (90:10:0.02 v/v/v) with 0.5 ml/min flow rate. Calibration curves were constructed by injecting standard mixture solutions at the seven concentrations of 2, 10, 50, 100, 250, 500, and 1000 ppm.

### Total carotenoid content

Total carotenoid content (TCC) was measured according to the procedure detailed by Ghazghazi et al.^11^. Briefly, 1 g powdered pericarp was mixed with acetone/methanol/petroleum ether (3:2:1 v/v/v) and kept at ambient temperature for 5 h in the dark. The extract was filtered with Whatman filter paper. The extract was partitioned with 50 ml diethyl ether and dried in a rotary at 35 °C. The dry extract was solved in 10 ml ethanol and mixed with 60% potassium hydroxide and boiled for 10 min. The extract was partitioned with diethyl ether. The diethyl ether fraction was evaporated and the dry extract was dissolved in 10 ml ethanol. The absorbance was recorded at 470 nm, using a spectrophotometer (Shimadzu double beam UV–visible spectrophotometer-1800, Japan). The date was expressed as mg β-carotene equivalents per 100 g of dry weight (mg β-CARE/100 g DW).

### Anthocyanins content

Evaluation of anthocyanin content was performed by the pH differential method^9^. Initially, 100 mg of dried powdered pericarp was added to 5 ml methanol/hydrochloric acid (1:1 v/v, pH = 2). Then 4 ml buffer solution (pH = 1) was mixed with 1 ml extract (pH = 4.5). The absorbance was calculated at wavelengths of 526 and 700 nm, using a spectrophotometer (Bio-Tek Instruments, Inc., USA). The anthocyanin content was calculated as follows equation:

$$\begin{aligned} & {\text{A}} = \left( {{\text{Absorbance of sample}}_{{526{\text{nm}}}} - {\text{Absorbance of sample}}_{{700{\text{nm}}}} } \right),pH = 1 \\ & {\text{B}} = \left( {{\text{Absorbance of sample}}_{{526{\text{nm}}}} - {\text{Absorbance of sample}}_{{700{\text{nm}}}} } \right),pH = 4.5 \\ & {\text{C}} = {\text{A}} - {\text{B}} \\ & {\text{Anthocyanins content}} = \left[ {\left( {{\text{C}}/{\text{MEC}}} \right) \times {\text{path length }}\left( {{\text{cm}}} \right)} \right] \times {\text{MW}}) \\ \end{aligned}$$

MEC is the molar extinction coefficient (26,900 L/Mcm for cyanidin 3-glucoside), MW is the molecular weight (449.2 g/M for cyanidin 3-glucoside).

Data expressed as mg cyanidin 3-glucoside/100 g of dry weight (mg C3G/100 g DW).

### Vitamin C assay

The AOAC^28^ method was used for vitamin C determination with the ascorbic acid standard. Initially, 1 g of powdered pericarp was mixed with 1 ml of metaphosphoric acid (3%) and centrifuged (Centrifuge Rotanta 460r, Hettich, Germany) at 1400 g for 10 min. The extract was titrated against 2,6-dichlorophenolindophenol dye solution (0.3%) to faint pink color. The amount of vitamin C was measured as follows formula:

${\text{Vitamin C }}\left( {{\text{mg/}}100{\text{ g DW}}} \right) = \left( {{\text{A}}/{\text{B}}} \right) \times 100.$ A is the (Standard concentration (mg/ml) × Titre value of the sample (ml) × 10, B is the Titre value of standard (ml) × Sample volume (ml) × Sample weight (mg).

### Total phenol and flavonoid content and antioxidant activity

Total phenol content (TPC) was determined as described previously by Singleton^29^. In summary, 25 μl pericarp methanolic extract (1000 ppm) and 125 μl Folin-Ciocalteu reagent, 100 μl sodium carbonate (7.5%) were taken in a test tube. The final volume was made up to 6 ml with distilled water. The solution was stored for 30 min in the dark. The absorbance was recorded at 765 nm using a spectrophotometer. The results are expressed as mg gallic acid equivalents (GAE)/per g of dry weight (mg GAE/g DW).

The total flavonoid content (TFC) was determined as described by Chang et al.^30^. Initially, 20 μl pericarp methanolic extract, 3.4 ml methanol (30%), 80 μl distilled water, 6 μl sodium nitrite (0.5 M), 6 μl aluminum chloride h (0.3 M) and 80 μl sodium hydroxide (1.0 M) was taken in a test tube and mixed well. The absorbance of the solution was determined against the reagent blank at 510 nm wavelength. The data were expressed as mg of rutin equivalents (RE) per g of dry weight (mg RE/g DW).

Antioxidant activity by the DPPH method was evaluated by Blois methods^31^. Briefly, 0.2 ml of methanolic extract and 4.0 ml DPPH solution was mixed into the test tube and incubated at room temperature for 20 min. The reduction of the DPPH radical was read using a spectrophotometer at 517 nm. Butylated hydroxytoluene was used as the control. The IC_50_ values were calculated as follows:

$${\text{DPPH scavenging effect }}\left( \% \right) = \left( {{\text{Abs}}_{0} {-}{\text{Abs}}_{1} /{\text{Abs}}_{0} } \right) \times 100.$$

Abs_0_ is the absorbance of the control, Abs_1_ is the absorbance of the sample.

### Statistical analysis

All the experiments in this study were performed in triplicate. The obtained results are expressed as the means ± standard deviation (SD). One-way analysis of variance (ANOVA) was used to calculate significant differences between studied ecotypes in terms of the traits measured with SPSS 16.0 (SPSS Inc., Chicago, IL, USA). A post-hoc test was run using Duncan’s test at *p* < 0.05. Cluster analysis was drawn using Euclidean distance coefficient and Ward’s method. The correlation between two sets of data was performed by multiple regression analysis, using the “stepwise” method of “linear regression analysis”. The Origin software version 2021 was applied to draw the heat map, and correlation plot, and biplot. Canonical correspondence analysis (CCA) was estimated with PAST 4.13 software.

## Results and discussion

### Morphological features

**Figure 1:**
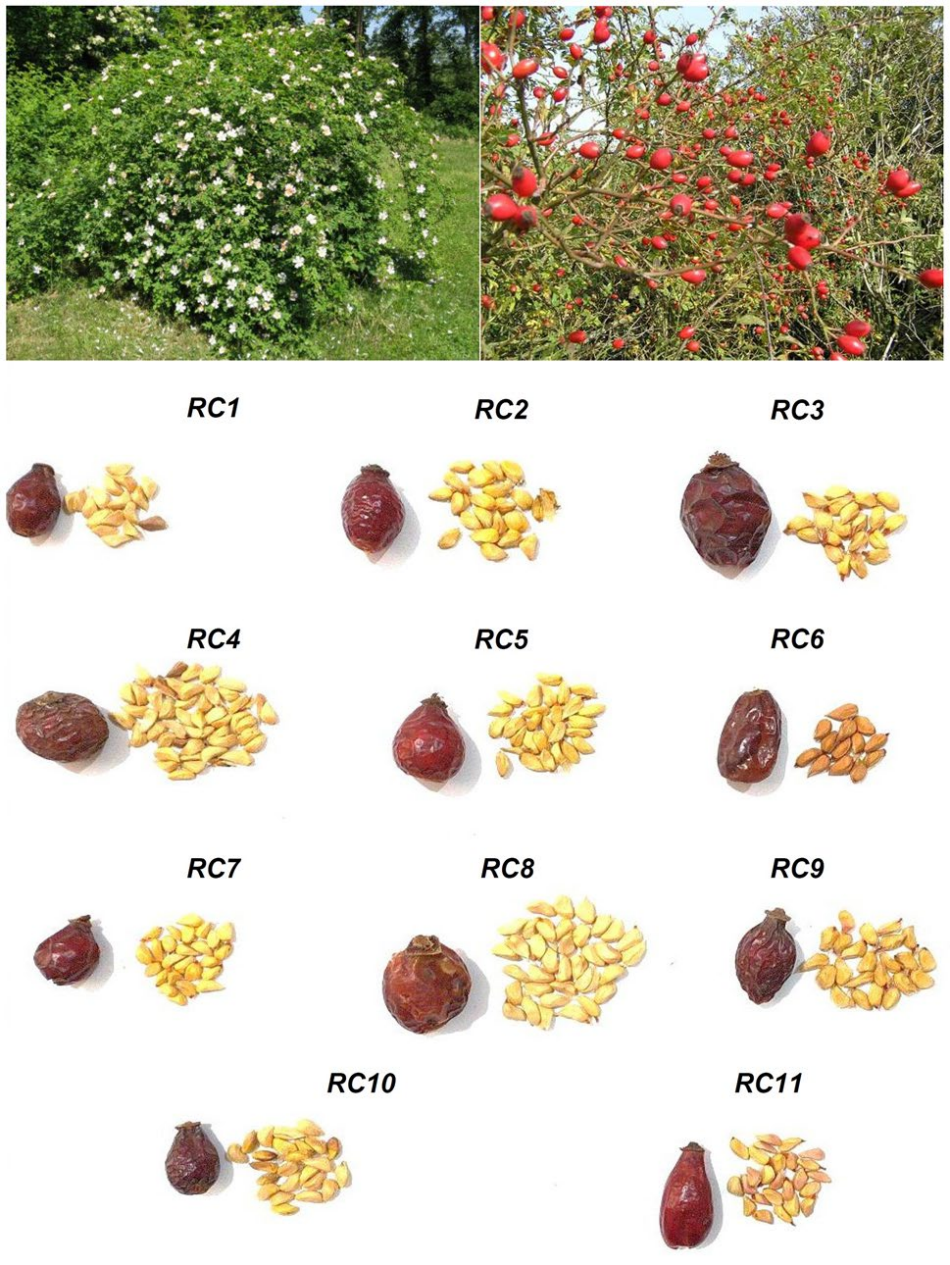
The pictures of shrub, fruit, and seed of *Rosa canina* ecotypes. For a detailed description of the plant ecotypes code, cf. Table 1.

The morphological traits of rose hips varied significantly among different RCEs (Table 2, Fig. 1). The coefficient of variation (CV%) was estimated at 21.13, 19.89, 33.92, 43.70, 31.92, and 46.57 for the traits of fruit length, fruit width, fruit weight, pericarp weight, number of seeds per fruit, and 1000 seed weight, respectively. A higher CV indicates a wider range of trait values, which offers more opportunities for selection^32^. The studied ecotypes differed in the morphological traits significantly (*p* < 0.05). The *RC8* ecotype exhibited the maximum fruit length (2.00 ± 0.073 cm) and width (1.40 ± 0.026 cm), while the *RC10* ecotype showed the minimum fruit length (0.97 ± 0.033 cm) and width (0.77 ± 0.005 cm). The lowest (0.46 ± 0.004) and highest (1.73 ± 0.023) fruit weight to g were recorded for the *RC1* and *RC8* ecotypes, respectively. The heaviest pericarps were 1.19 ± 0.044 g produced by the *RC3* ecotype and the lightest were 0.21 ± 0.003 g produced by the *RC1* ecotype. The *RC4* ecotype was produced the highest number of seeds per fruit (on average, 43.67 seeds), and *RC6* ecotype produced the lowest number (on average, 14.42 seeds). The minimum and maximum of 1000 seed weights were 97.92 ± 2.56 and 596.85 ± 4.55 g related to *RC1* and *RC3* ecotypes, respectively. The diversity of rose hip traits among RCEs has previously been reported by other researchers so far^33,34^. Guo et al.^35^ indicated that the wild edible fruits of the plant have more unique genetic diversity and genetic. Clearly, wild species can enhance the genetic diversity of crops^36^. The recurrent propagation of the wild fruit seeds in nature increases their genetic diversity^37^.

**Table 2:** Comparison of different morphological traits among *Rosa canina* ecotypes.

No.	Fruit length (cm)	Fruit width (cm)	Fruit weight (g)	Pericarp weight (g)	Seed number per fruit	1000 seed weight (g)
1	1.60 ± 0.022f	1.30 ± 0.112c	0.46 ± 0.004k	0.21 ± 0.003j	22.53 ± 1.43e	97.92 ± 2.56j
2	1.23 ± 0.040i	1.20 ± 0.056e	1.13 ± 0.010e	0.62 ± 0.027e	22.31 ± 0.79e	280.94 ± 2.14d
3	1.90 ± 0.018d	1.14 ± 0.078f	1.59 ± 0.041b	1.19 ± 0.044a	20.45 ± 0.48g	596.85 ± 4.55a
4	1.22 ± 0.049i	1.25 ± 0.004d	1.34 ± 0.003d	0.69 ± 0.021d	43.67 ± 1.12a	157.53 ± 3.20i
5	1.55 ± 0.112g	1.38 ± 0.033b	1.54 ± 0.101c	1.13 ± 0.028b	30.53 ± 1.90c	377.99 ± 1.56b
6	1.92 ± 0.005b	1.02 ± 0.015g	0.76 ± 0.013j	0.52 ± 0.019g	14.42 ± 0.16i	354.60 ± 0.79c
7	1.34 ± 0.091h	0.91 ± 0.004h	0.93 ± 0.005h	0.57 ± 0.014f	21.65 ± 0.44f	268.11 ± 4.20e
8	2.00 ± 0.073a	1.40 ± 0.026a	1.73 ± 0.023a	0.76 ± 0.006c	35.98 ± 0.98b	218.41 ± 0.68g
9	1.80 ± 0.150e	0.90 ± 0.013h	1.04 ± 0.002f	0.47 ± 0.012h	20.32 ± 1.22h	226.06 ± 3.47f
10	0.97 ± 0.033j	0.77 ± 0.005j	0.95 ± 0.001g	0.45 ± 0.008i	20.64 ± 1.13g	225.70 ± 1.09f
11	1.91 ± 0.001c	0.80 ± 0.002i	0.78 ± 0.004i	0.45 ± 0.010i	22.86 ± 0.58d	205.61 ± 2.63h
CV (%)	21.13	19.89	33.92	43.70	31.92	46.57

Data are mean ± standard deviation (n = 3).

Values followed by the same letter within each column are significantly different (*p* < 0.05).

### Fatty acid profile

The knowledge about the seed oils and fatty acid profile of rose hip is extremely rare. The results of the GC analysis of some studied RCEs are presented in Table 3. The seed oil was ranged from 8.08 ± 0.17% to 16.91 ± 0.35%. The highest was observed in the *RC5* ecotype. Javanmard et al.^38^ was reported 8.09 ± 0.15% to 11.43 ± 0.31% of the seed oil content in five RCEs.

**Table 3:** Fatty acid profiles of the studied *Rosa canina* ecotypes.

FA (%)	*RC1*	*RC2*	*RC3*	*RC4*	*RC5*	*RC6*	*RC7*	*RC8*	*RC9*	*RC10*	*RC11*
Oil yield	11.81 ± 0.56c	10.19 ± 0.68g	16.72 ± 0.97b	11.12 ± 0.52d	16.91 ± 0.35a	8.08 ± 0.17h	10.31 ± 0.78g	10.41 ± 0.31f.	11.01 ± 0.15d	11.68 ± 0.94c	10.70 ± 0.45e
C12:1	0.31 ± 0.01f	0.30 ± 0.00f	0.18 ± 0.01g	0.47 ± 0.01c	0.46 ± 0.06c	0.33 ± 0.02e	0.40 ± 0.05d	0.51 ± 0.01b	0.71 ± 0.01a	0.33 ± 0.02e	0.52 ± 0.03b
C14:0	0.19 ± 0.01j	0.21 ± 0.01i	0.63 ± 0.01d	4.32 ± 0.06a	0.99 ± 0.04c	0.54 ± 0.01g	2.25 ± 0.08b	0.55 ± 0.01f	0.56 ± 0.07f	0.26 ± 0.01h	0.58 ± 0.01e
C16:0	6.11 ± 0.22j	7.64 ± 0.45e	6.82 ± 0.26g	9.10 ± 0.36c	6.05 ± 0.21k	7.54 ± 0.04f	6.51 ± 0.06i	9.03 ± 0.53d	12.08 ± 0.17a	6.67 ± 0.05h	10.11 ± 0.16b
C18:0	1.40 ± 0.01e	1.00 ± 0.00f	0.78 ± 0.01h	1.71 ± 0.04c	1.75 ± 0.03c	0.71 ± 0.05i	2.58 ± 0.01a	0.97 ± 0.07g	1.61 ± 0.01d	1.83 ± 0.01b	0.09 ± 0.00i
C18:1c	11.18 ± 0.00d	9.63 ± 0.06g	19.25 ± 0.25a	13.46 ± 0.71c	1.23 ± 0.01i	0.80 ± 0.01j	2.01 ± 0.05h	10.47 ± 0.16f	14.05 ± 0.02b	12.69 ± 0.13d	11.05 ± 0.28e
C18:1t	3.58 ± 0.01i	5.29 ± 0.11f	9.74 ± 0.17e	12.09 ± 0.07d	18.03 ± 0.56c	27.88 ± 0.73a	23.42 ± 0.97b	4.82 ± 0.04g	1.99 ± 0.01k	4.56 ± 0.01h	3.49 ± 0.09j
C18:2	49.49 ± 1.29b	47.44 ± 1.06c	40.10 ± 1.90i	35.41 ± 0.78k	49.59 ± 0.96a	41.00 ± 1.10g	42.89 ± 1.33e	40.77 ± 1.50h	42.21 ± 0.44f	45.09 ± 0.36d	39.37 ± 0.56j
C20:0	23.84 ± 0.32d	25.35 ± 0.41a	21.15 ± 0.12f	21.31 ± 0.63e	17.67 ± 0.06j	19.47 ± 0.37g	18.10 ± 0.17i	25.36 ± 0.54a	19.85 ± 0.43g	24.64 ± 1.06c	24.82 ± 0.81b
C20:4	0.01 ± 0.00j	1.23 ± 0.01c	0.35 ± 0.01h	2.03 ± 0.01a	1.00 ± 0.00d	0.83 ± 0.00e	1.74 ± 0.01b	0.10 ± 0.00i	0.78 ± 0.01f	0.72 ± 0.01g	0.79 ± 0.01f
Others	3.89 ± 0.03d	1.91 ± 0.01f	1.00 ± 0.00j	0.10 ± 0.00i	3.23 ± 0.02e	0.90 ± 0.01h	0.10 ± 0.00i	7.42 ± 0.15b	6.16 ± 0.24c	3.21 ± 0.03e	9.18 ± 0.08a
SFA	**31.54 ± 0.21g**	**34.20 ± 0.14d**	**29.38 ± 0.12h**	**36.44 ± 0.18a**	**26.46 ± 0.25i**	**28.26 ± 0.30h**	**29.44 ± 0.19g**	**35.91 ± 0.17c**	**34.10 ± 0.17e**	**33.40 ± 0.21f**	**35.60 ± 0.08b**
USFA	**64.57 ± 0.67d**	**63.89 ± 1.01e**	**69.62 ± 1.31b**	**63.46 ± 0.58f**	**70.31 ± 0.91b**	**70.84 ± 1.11a**	**70.46 ± 1.27c**	**56.67 ± 1.41h**	**58.74 ± 0.36g**	**63.39 ± 0.50e**	**55.22 ± 0.53i**
MUFA	**15.07 ± 0.21g**	**15.22 ± 0.11f**	**29.17 ± 0.13a**	**26.02 ± 0.05b**	**19.72 ± 0.01c**	**29.01 ± 0.15a**	**25.83 ± 0.10b**	**15.80 ± 0.09f**	**16.75 ± 0.14e**	**17.58 ± 0.18d**	**15.06 ± 0.22g**
PUFA	**49.50 ± 1.15b**	**48.67 ± 1.04c**	**40.45 ± 1.91i**	**37.44 ± 0.86k**	**50.59 ± 0.81a**	**41.83 ± 0.95g**	**44.63 ± 1.06e**	**40.87 ± 1.26h**	**42.99 ± 0.64f**	**45.81 ± 0.51d**	**40.16 ± 0.63j**

Data are mean ± standard deviation (n = 3).

Values followed by the same letter within each row are significantly different (*p* < 0.05).

*FA* fatty acids, *SFA* saturated fatty acids, *USFA* unsaturated fatty acids, *MUFA* monounsaturated fatty acids, *PUFA* polyunsaturated fatty acids.

Significant values are in bold.

**Figure 2:**
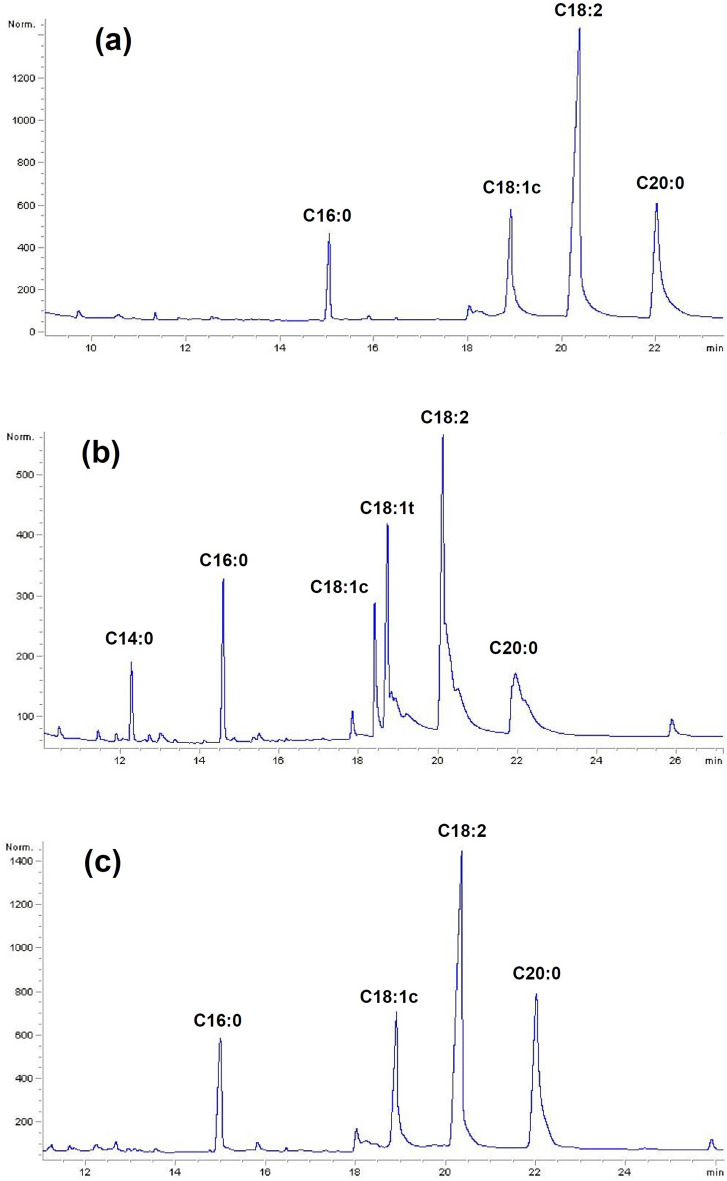
A typical chromatogram of the fatty acids from *RC1* (**a**), *RC4* (**b**), and *RC11* (**c**) samples of *Rosa canina* ecotypes.

Nine fatty acids were detected in the studied samples that represented 90.82 ± 0.44–99.90 ± 0.18% of the seed oil. The SFA and USFA ranged from 26.46 ± 0.025% to 36.44 ± 0.18% and from 55.22 ± 0.53% to 70.84 ± 1.11%, respectively. The *RC5* and *RC4* ecotypes had the lowest and highest SFA percentage, respectively. The maximum of USFA was obtained from the *RC6* ecotype. Monounsaturated fatty acids (MUFA) varied from 15.06 ± 0.22% to 29.01 ± 0.15%, whereas polyunsaturated fatty acids (PUFA) varied from 37.44 ± 0.86% to 50.59 ± 0.81%. Eicosanoic acid (from 17.67 ± 0.06% to 25.36 ± 0.54%) was the main SFA in the studied ecotypes. It was the most abundant in the *RC8* ecotype. Oleic acid (0.80 ± 0.01% to 19.25 ± 0.25%) was also primary PUFA that the highest level were found in *RC3* ecotypes. Figure 2 displays the typical chromatogram of the fatty acids of several ecotypes.

Popovic-Djordjevic et al.^26^ reported linoleic acid (24.53 ± 0.96% to 46.68 ± 1.34%) and palmitic acid (9.77 ± 0.21% to 35.68 ± 1.22%) in two varieties and six RCEs from Serbia. Kulaitiene et al.^39^ stated that environmental conditions, genotype, and extraction method were effective on the oil content of *R. canina* seeds as well as fatty acids compounds. Due to the importance of USFA for human health, the high oil content, and USFA in the seeds of the studied ecotypes, the significance of the studied *R. canina* is evident.

### Total phenol and flavonoid content and antioxidant activity

**Figure 3:**
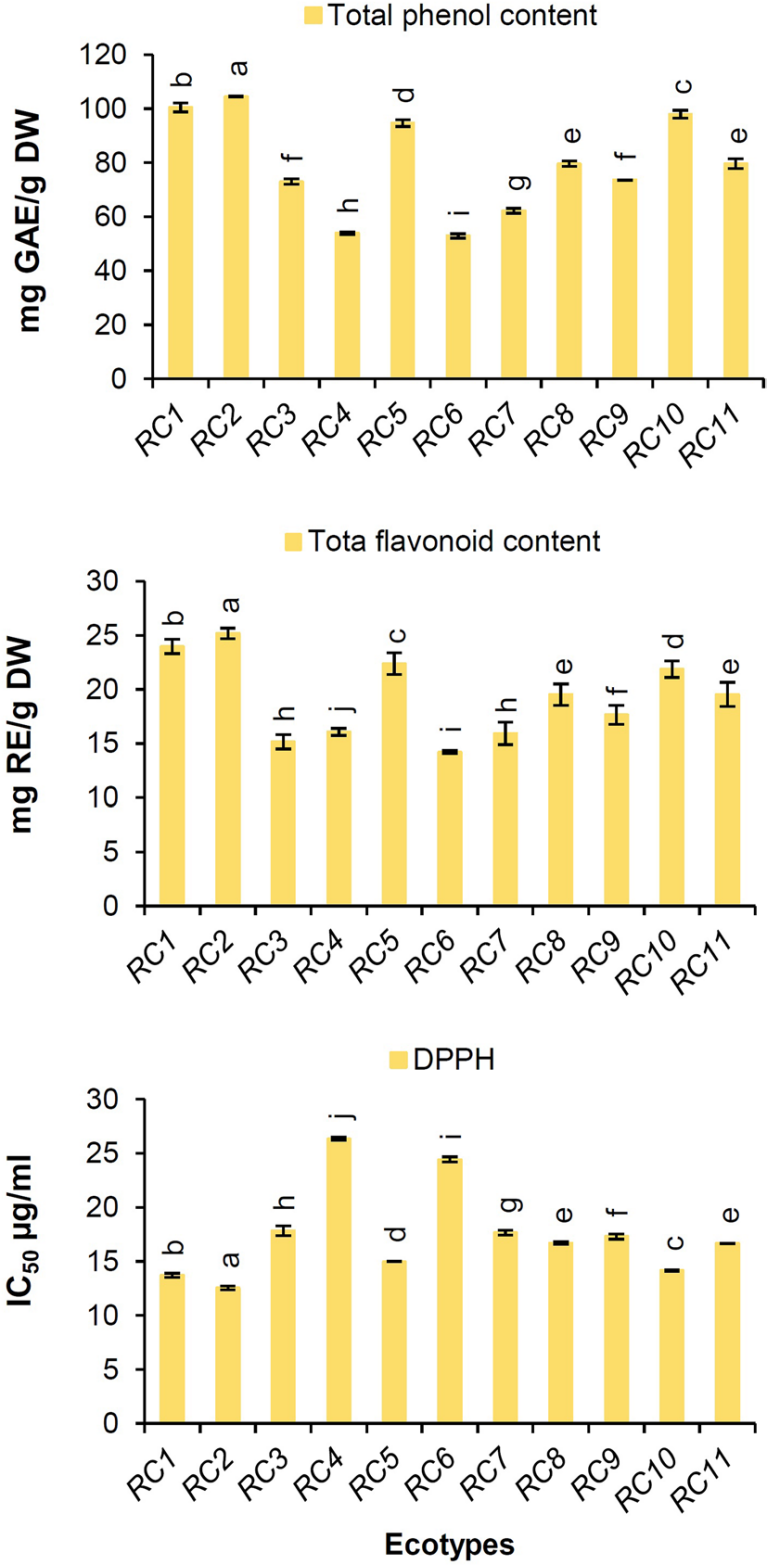
Histogram of total phenol content, total flavonoid content, and antioxidant activity among *Rosa canina* ecotypes.

Figure 3 depicts the range of TPC and TFC for all studied RCEs. The ecotypes was differed in the TPC and TFC significantly (*p* < 0.05). The TPC was in the range of 52.87 ± 0.82–104.42 ± 0.23 mg GAE/g DW, and the TFC was in the range of 14.20 ± 0.12–25.18 ± 0.47 mg RE/g DW. The lowest TPC and TFC were obtained in *RC6* and the highest was found in *RC2*. According to previous studies, the TPC of fresh fruits from *Rosa* species in different regions of the world was from 177 to 816 mg GAE/100 g FW^40,41^. Medveckiene et al.^42^ were obtained also the TPC of fresh fruits in the range of 150–299 mg GAE/100 g DW in various *Rosa* species. Jemaa et al.^43^ reported the TFC in the *R. canina* rose hip methanolic extract was 2.64 mg RE/g. Nadpal et al.’s^44^ study on *R. canina* and *R. arvensis* Huds. species revealed that the TFC was from 0.63 to 1.48 mg RE/g.

The studied RCEs was differed in TPC and TFC significantly. Polyphenolic compounds can capture free radicals due to their chemical structure and form complexes with metal ions. Therefore, these compounds showed good antioxidant activity. There are some methods to produce polyphenolic compounds in plants and various mechanisms for their distribution across different plant structures. Genetics, environmental conditions, climatic conditions, and the solvent used in extraction are some factors that influence the level of phenolic and flavonoid compounds derived from plants^45^.

The lower the half maximal inhibitory concentrate (IC_50_) is in the rose hip extract, the higher the antioxidant activity will be. The highest IC_50_ (μg/ml) was found in *RC4* (26.33 ± 0.13) and *RC6* (24.41 ± 0.24) ecotypes and the minimum was found in *RC2* (12.54 ± 0.18), *RC1* (13.70 ± 0.19), and *RC10* (14.15 ± 0.08) ecotypes (Fig. 3).

The studied ecotypes exhibited significant diversity in antioxidant activity, which is consistent with Okatan et al.’s^37^ study on RCEs. Also, a significant diversity of antioxidant activity was detected among Romanian RCEs^46^. Shameh et al.^47^ were found a significant difference (*p* < 0.05) in the rose hip antioxidant activity between *R. hemisphaerica* Herrm. and *R. canina* ecotypes. They attributed this difference to genetics, geographical region, climatic conditions, and the type of sample used. Roby et al.^48^ have shown the extracts that are rich in phenolic compounds have much stronger antioxidant effects than extracts without these compounds. This study showed similarly that ecotypes containing more phenolic compounds had stronger antioxidant activity.

### Total carotenoids, and anthocyanin contents, and vitamin C contents

**Figure 4:**
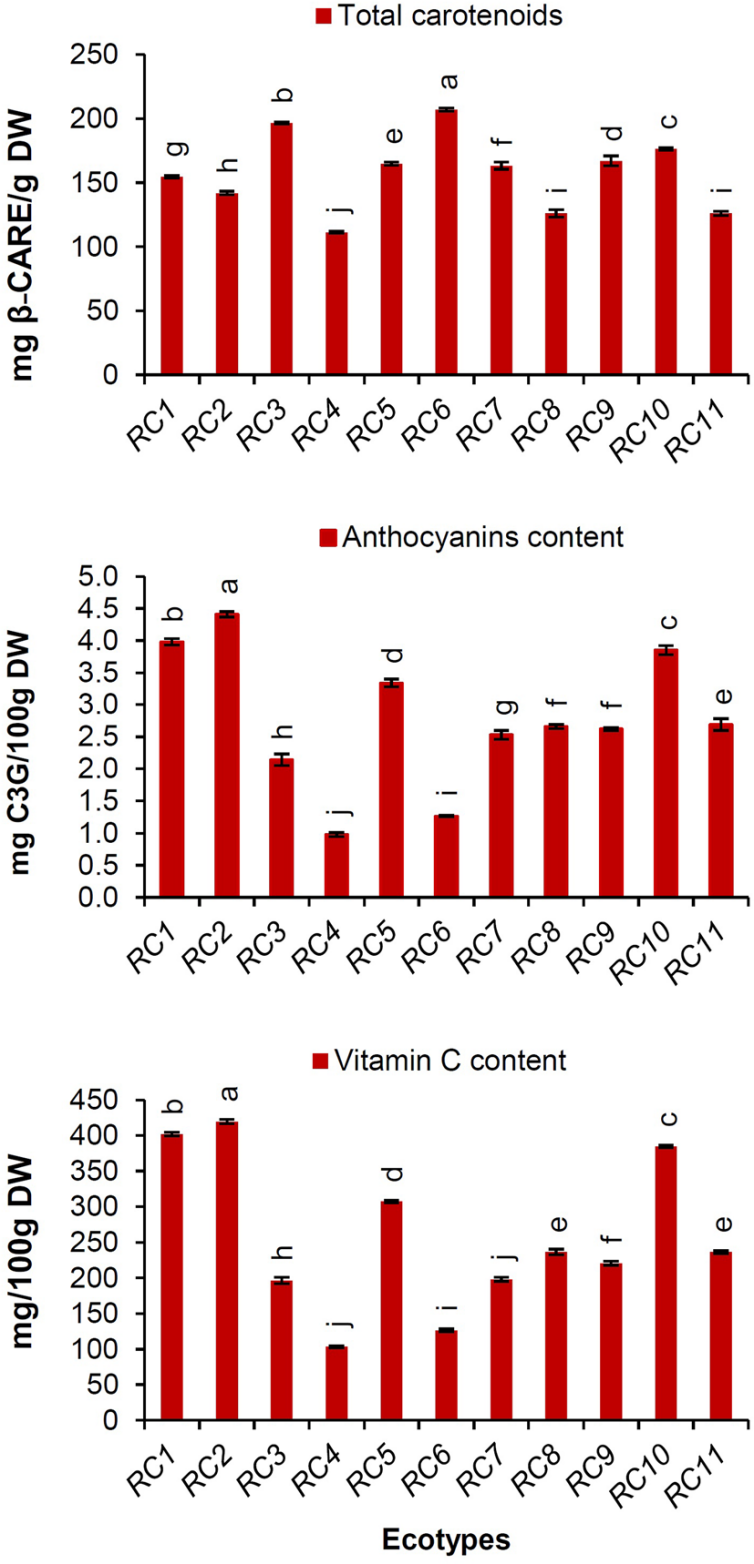
Histogram of pigments and vitamin C content among the studied *Rosa canina* ecotypes.

A wide range of diversity in the TCC was detected among ecotypes (Fig. 4). The TCC was varied from the minimum value of 111.22 ± 0.78 mg β-CARE/g DW in *RC4* to the maximum value of 206.98 ± 1.25 mg β-CARE/g DW in *RC6*.

The red-to-blue color of the fruits is caused by their anthocyanins, which have strong anti-inflammatory and antioxidant activities. The studied ecotypes showed significant differences in the anthocyanin content (*p* < 0.05). The anthocyan in content varied from 0.98 ± 0.03 to 4.41 ± 0.04 mg C3G/100 g DW (Fig. 4). The *RC2*, *RC1*, and *RC10* ecotypes had the highest anthocyanin contents of 4.41 ± 0.04, 3.98 ± 0.05, and 3.85 ± 0.05 mg C3G/100 g DW, respectively. However, the lowest content (0.98 ± 0.03 mg C3G/100 g DW) was obtained in *RC4*. The level of anthocyanin in *R. canina* has been reported to be 2.75, 2.82, and 2.94 mg CGE/100 g by Murathan et al.^49^, Yildiz and Alpaslan^50^, and Fascella et al.^1^, respectively. Szmagara et al.^51^ estimated the anthocyanin content of dried *R. sweginzowii* Koehne rose hips at 0.43–7.4 mg CGE/100 g.

The high antioxidant activity of rose hip is related to its high level of vitamin C^52^. The RCEs differed significantly in the compound (*p* < 0.05). The vitamin C content varied from 103.51 ± 1.24 to 419.70 ± 3.12 mg/100 g DW in different ecotypes. These values were observed in *RC4* and *RC2* ecotypes, respectively (Fig. 4). Kayahan et al.^52^ were reported vitamin C content of *R. corymbifera* Borkh., *R. rugosa* (Thunb.), *R. alba* L., and *R. canina* genotypes in the range of 180 to 965 mg/100 g. Our results are consistent with the reports of Erogul and Ogus^53^ and Bilgin et al.^54^. Kayahan et al.^52^ were mentioned that genotype is the key factor affecting the vitamin C content of *Rosa* genotypes.

### Phenolic acids

**Figure 5:**
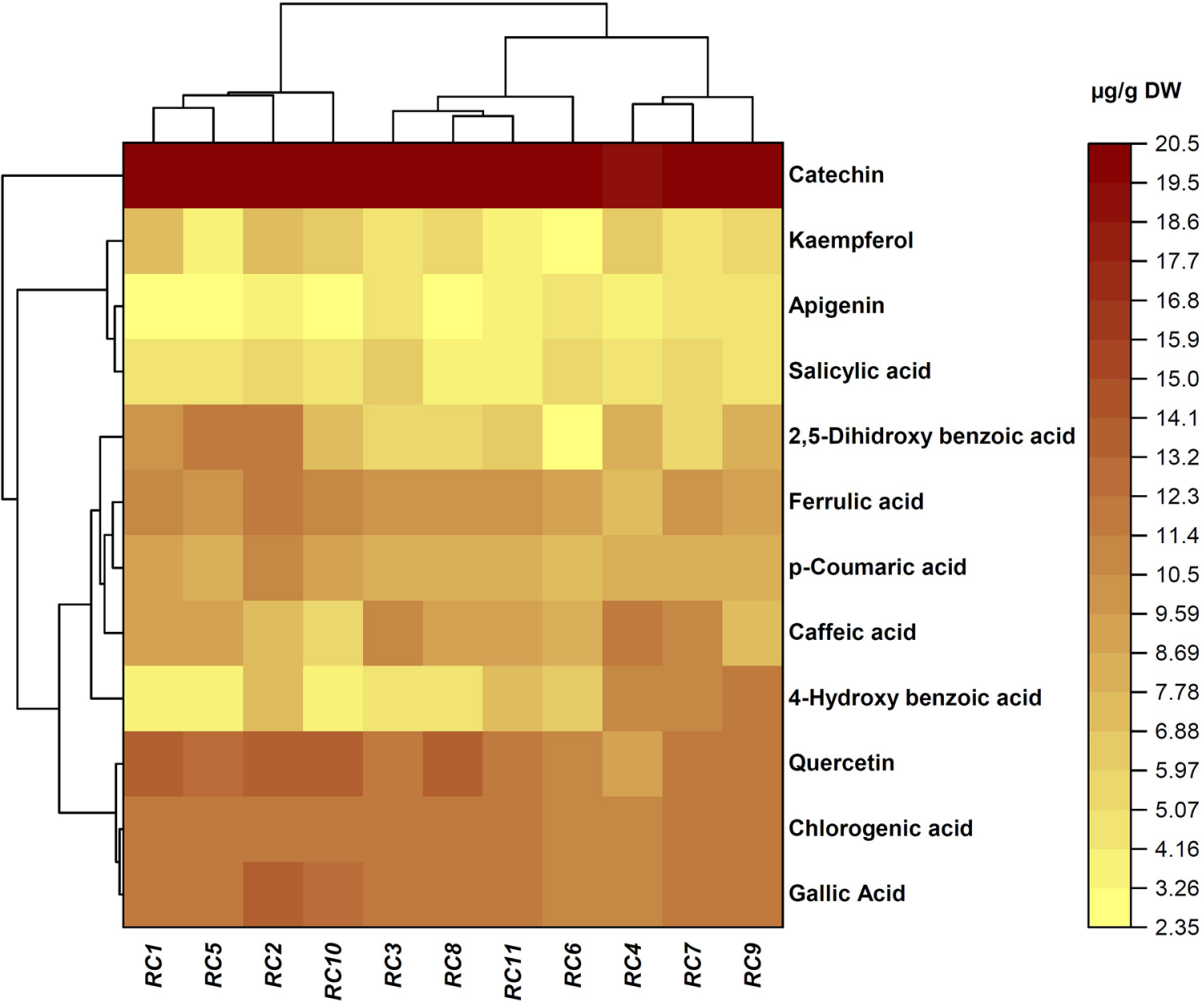
Heat map of the phenolic compound profiles of *Rosa canina* ecotypes. Mean values refer to colors from minimum displayed in yellow to maximum represented with crimson.

The main phenolic compounds in the rose hips flesh included catechin, quercetin, gallic, chlorogenic, ferulic, *p*-coumaric, caffeic, 2,5-dihydroxy benzoic, and 4-hydroxy benzoic acid, kaempferol, salicylic acid, and apigenin (Fig. 5). In *RC2* ecotype, the highest catechin, quercetin, gallic acid, chlorogenic acid, ferulic acid, *p*-coumaric acid, and kaempferol content were obtained 20.42 ± 0.47, 13.82 ± 0.04, 13.52 ± 0.21, 12.03 ± 0.13, 11.42 ± 0.12, 10.92 ± 0.45, and 7.32 ± 0.19 µg/g DW, respectively. The *RC4* ecotype had the highest level (11.43 ± 0.14 µg/g DW) of caffeic acid whereas the lowest level (5.12 ± 0.03 µg/g DW) was observed in the *RC10* ecotype. The 2,5-dihydroxy benzoic and 4-hydroxy benzoic acid content varied from 3.17 ± 0.019 (*RC6*) to 11.81 ± 0.02 µg/g DW (*RC5*) and from 3.98 ± 0.01 (*RC5*) to 11.86 ± 0.09 µg/g DW (*RC9*), reflecting the high diversity of the studied ecotypes. Salicylic acid content was in the range of 4.13 ± 0.05–5.98 ± 0.07 µg/g DW. The minimum and maximum levels of the salicylic acid were detected in *RC8* and *RC3* ecotypes, respectively.

Ozturk et al.^55^ reported that protocatechuic (1.4 mg/100 g), vanillic acid (6.9 mg/100 g), chlorogenic acid (8.5 mg/100 g), *p*–coumaric acid (24.9 mg/100 g), and ferulic acid (23.9 mg/100 g), and catechin (3.1 mg/100 g), were the most abundant phenolic compounds in *R. canina*. Shameh et al.^47^ reported chlorogenic and gallic acid as the most abundant phenolic compounds in rose hips from Iran. Chlorogenic, gallic, *p*–coumaric, and caffeic acid were the main phenolic compounds in five *rose* species grown in Turkey^22^. The catechin content was obtained in the range of 2.37 to 7.83 µg/g in rose hips species by Nadpal et al.^15^. The quantitative and qualitative differences in phenolic compounds among different RCEs may be related to different genetic and environmental factors (e.g. nutrition, light, and temperature), and maturity stages of rose hip^56^.

### Correlation, cluster, and factor analysis

**Figure 6:**
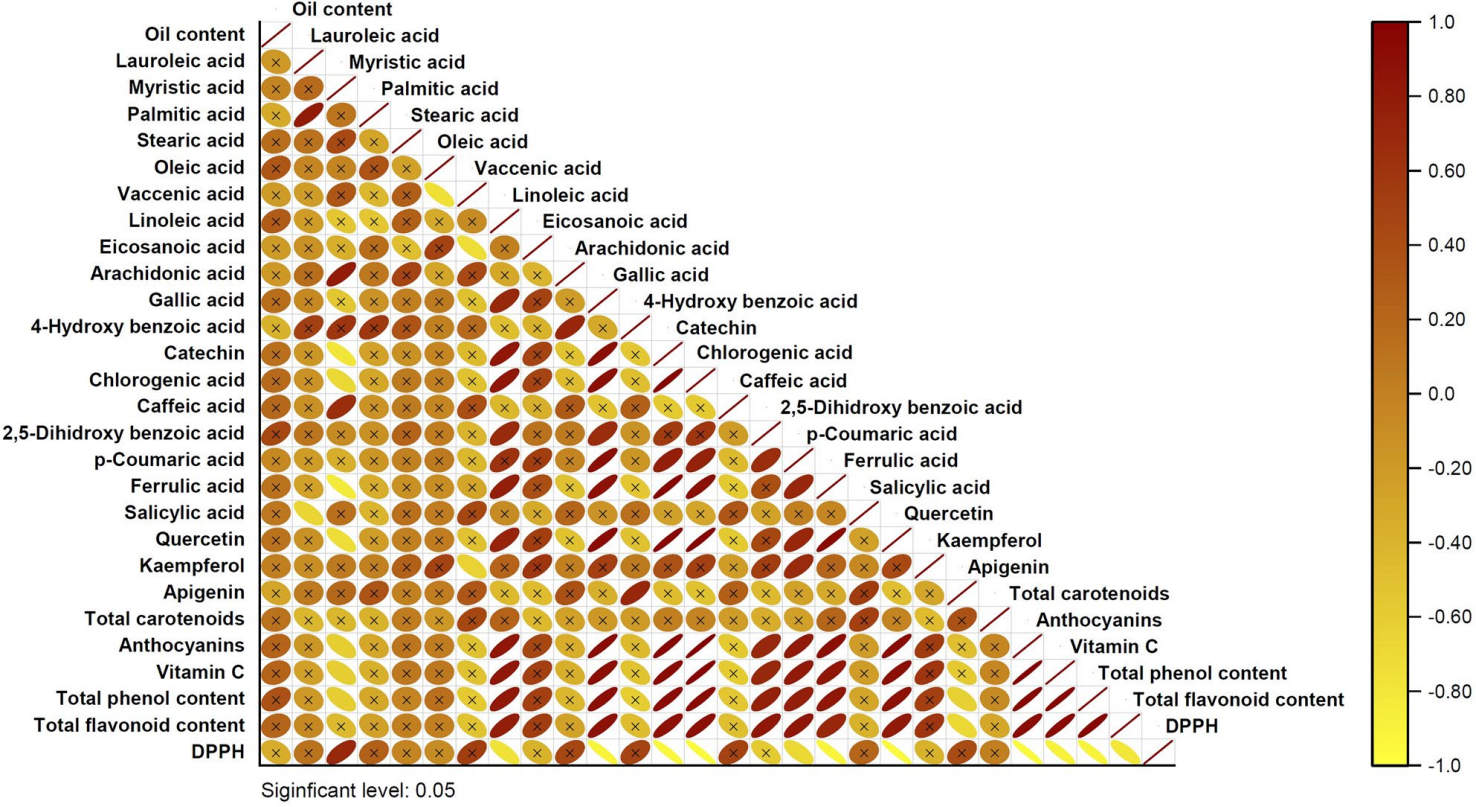
Linear correlation between the phytochemical traits and antioxidant activity. Significant difference in 5% level.

The relationships between phytochemical traits were calculated by Pearson’s correlation test and displayed by a heat map (Fig. 6). Vitamin C and anthocyanins had positive and significant correlations with linoleic acid, gallic acid, catechin, chlorogenic acid, 2,5–dihydroxy benzoic acid, *p*–coumaric acid, ferrulic acid, and quercetin had negative and significant correlations with myristic acid. The seed oil content and TCC, two important economic traits, had no significant correlations with other phytochemical traits. The awareness of the relationships between the traits is important for selection in breeding works.

**Figure 7:**
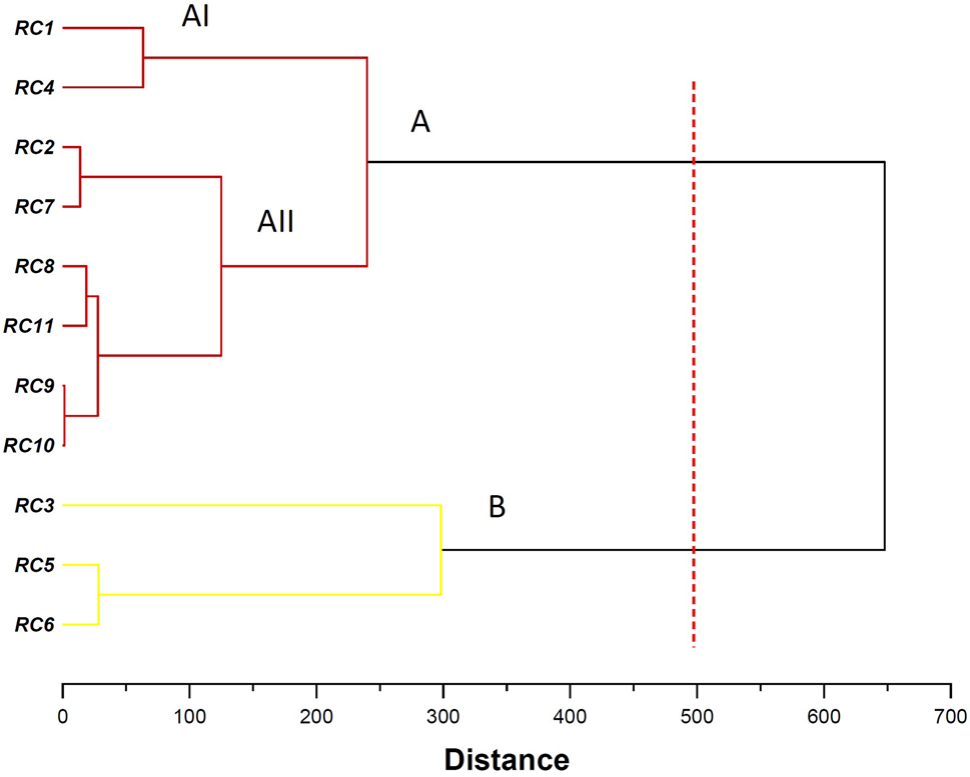
Ward cluster analysis of *Rosa canina* ecotypes based on morphological traits using Euclidean distances.

Cluster analysis is a major method to group individuals, populations, and ecotypes in terms of various traits. The ecotypes were put into two main groups according to cluster based on phytochemical characters (Fig. 7). The first cluster (A) had two sub-clusters, the first (AI) including two ecotypes and the second (AII) including six ecotypes. The second cluster (B) contained three ecotypes. The studied ecotypes exhibited significant diversity in phytochemical traits.

According to the results of principal component analysis (PCA), the first to seventh factors accounted for 93.53 percent of the total variance (Table 4). Myristic, linoleic, vaccenic, eicosanoic, gallic, 4–hydroxy benzoic, chlorogenic, caffeic, 2,5–dihydroxy benzoic, *p*–coumaric, and ferulic acid, catechin, quercetin, apigenin, anthocyanins, vitamin C, TPC, TFC, and DPPH were the traits in the first component that had the highest impact factors and accounted for 40.85 percent of the total variance. The greatest impact factors in the second component were related to 1000 seed weight, lauroleic, palmitic, and salicylic acid, kaempferol, and TCC, which captured 14.72 percent of the total variance.

**Table 4:** Eigenvalues of the principal component axes from the multiple regression analysis of the studied parameters in *Rosa canina* ecotypes.

Traits	Component						
	I	II	III	IV	V	VI	VII
Fruit length	− 0.260	0.295	− 0.342	− 0.679**	− 0.290	0.110	0.232
Fruit width	0.108	0.054	0.710**	− 0.278	− 0.246	− 0.194	0.320
Fruit weight	− 0.186	0.144	0.649**	− 0.473	0.089	0.307	0.170
Pericarp weight	− 0.239	0.513**	0.653**	− 0.324	0.081	0.306	0.093
Seed number per fruit	− 0.221	− 0.569**	0.713**	− 0.257	− 0.152	− 0.088	0.058
1000 seed weight	− 0.232	0.843**	0.173	− 0.194	0.284	0.240	0.062
Oil content	0.200	0.312	0.634**	− 0.259	0.078	0.310	− 0.472
Lauroleic acid	− 0.177	− 0.611**	− 0.193	− 0.140	− 0.411	0.598**	0.002
Myristic acid	− 0.681**	− 0.445	0.488	0.277	0.070	− 0.081	− 0.029
Palmitic acid	− 0.279	− 0.587**	− 0.421	− 0.385	0.063	0.462	0.129
Stearic acid	− 0.039	− 0.126	0.375	0.700**	− 0.050	0.220	− 0.319
Oleic acid	0.081	− 0.253	0.014	− 0.551**	0.700**	0.028	− 0.340
Vaccenic acid	− 0.570**	0.494	0.093	0.503**	− 0.326	− 0.135	0.194
Linoleic acid	0.805**	0.298	0.136	0.332	− 0.270	0.091	− 0.041
Eicosanoic acid	0.565**	− 0.381	− 0.184	− 0.431	0.285	− 0.416	0.128
Arachidonic acid	− 0.472	− 0.332	0.285	0.627**	0.139	0.153	0.190
Gallic acid	0.911**	− 0.047	0.068	0.061	0.135	0.226	0.264
4-Hydroxy benzoic acid	− 0.559**	− 0.464	− 0.172	0.405	0.235	0.404	0.165
Catechin	0.965**	0.141	− 0.102	− 0.020	− 0.093	0.057	0.143
Chlorogenic acid	0.979**	0.025	− 0.064	0.091	− 0.051	0.029	− 0.029
Caffeic acid	− 0.604**	0.038	0.469	− 0.068	− 0.006	− 0.188	0.175
2,5-Dihidroxy benzoic acid	0.651**	− 0.260	0.474	0.162	− 0.065	0.275	− 0.006
*p*-Coumaric acid	0.803**	− 0.159	0.077	0.229	0.288	− 0.013	0.409
Ferrulic acid	0.881**	0.352	− 0.148	0.019	− 0.013	0.101	0.208
Salicylic acid	− 0.292	0.587**	0.209	0.255	0.590**	− 0.094	0.244
Quercetin	0.944**	0.076	− 0.105	− 0.035	− 0.018	0.061	0.076
Kaempferol	0.542**	− 0.556**	0.177	0.146	0.485	− 0.199	− 0.066
Apigenin	− 0.582**	0.189	− 0.388	0.227	0.436	0.355	0.230
Total carotenoids	− 0.085	0.840**	− 0.315	0.204	0.068	0.070	− 0.266
Anthocyanins	0.981**	0.018	0.033	0.144	0.038	0.097	− 0.009
Vitamin C	0.984**	0.011	0.055	0.132	0.053	− 0.034	− 0.067
Total phenol content	0.976**	0.020	0.149	− 0.048	0.005	0.048	− 0.078
Total flavonoid content	0.935**	− 0.197	0.193	0.052	− 0.110	− 0.031	0.059
DPPH	− 0.930**	− 0.130	− 0.006	0.016	− 0.038	− 0.235	0.025
Eigenvalue	13.89	5.00	4.06	3.50	2.19	1.84	1.32
% of variance	40.85	14.72	11.96	10.30	6.43	5.41	3.88
Cumulative %	40.85	55.57	67.53	77.83	84.26	89.68	93.56

**Eigenvalues significant > 0.50.

**Figure 8:**
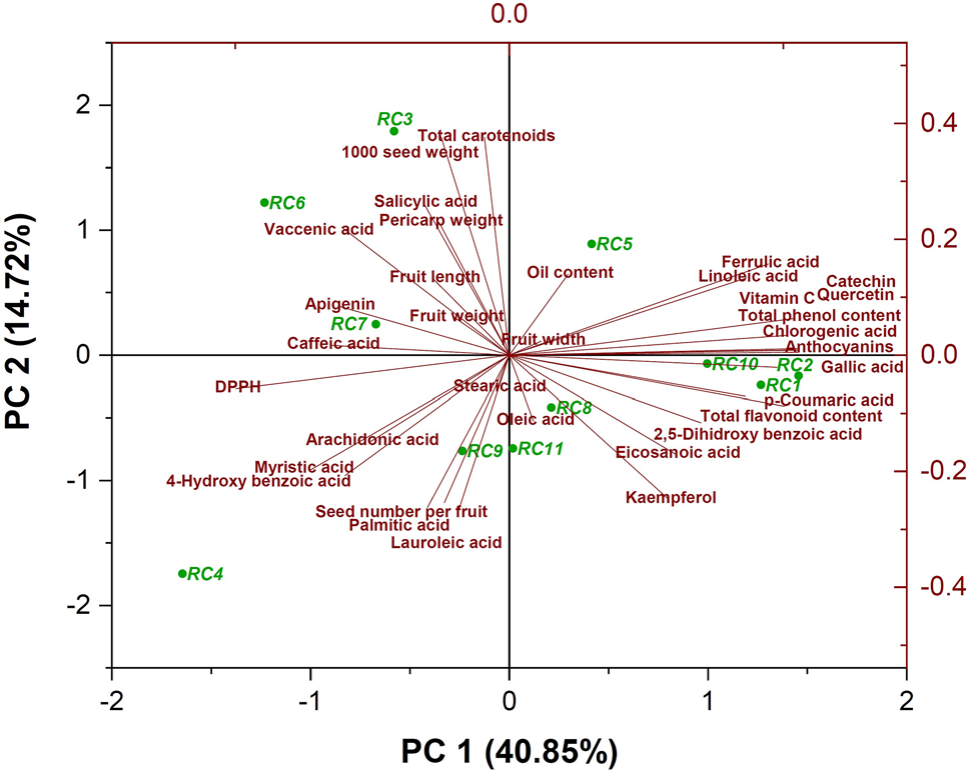
Biplot analysis of *Rosa canina* ecotypes based on phytochemical and morphological traits.

According to Fig. 8, *RC5* ecotype was placed in the first zone of the biplot, which included positive values of both components. It was related to the traits of oil content, ferulic acid, linoleic acid, catechin, vitamin C, quercetin, TPC, chlorogenic acid, and anthocyanin. The second zone of the biplot, hosted *RC3*, *RC6*, and *RC7* ecotypes, that included negative and positive values of the first and second components, respectively. The group was related to the traits of vaccenic acid, 1000 seed weight, TCC, salicylic acid, pericarp weight, fruit length, and weight, apigenin, and caffeic acid. The third zone of the biplot, in which the values of both components were negative, contained *RC4* and *RC9* ecotypes. These ecotypes were related to the traits of DPPH, arachidonic acid, myristic acid, 4–hydroxy benzoic acid, seed number per fruit, palmitic acid, and lauroleic acid, and stearic acid. Finally, the fourth zone contained *RC10*, *RC2*, *RC1*, *RC8*, and *RC11* ecotypes, that were related to the traits of TFC, kaempferol, and gallic, *p*–coumaric, 2,4–dihydroxy benzoic, eicosanoic, and oleic acid. The results of comparing the cluster analysis and PCA revealed similarities between them. The ecotypes which were found by the cluster analysis to be superior in phytochemical traits were put in the same group by PCA.

### Multiple regression analysis

The multiple regression analysis showed that the correlation between phenotypic data (as independent traits) and phytochemical traits (as dependent traits) was significant (*p* < 0.05; *p* < 0.01). The morphological trait of 1000 seed weight was related to palmitic acid (*β* = − 599), arachidonic acid (*β* = 679), and TCC (*β* = 449), whereas pericarp weight was correlated with vaccenic acid (*β* = 642), salicylic acid (*β* = 349), and TCC (*β* = − 620). In addition, the two variables of fruit length and fruit weight had significant relationships with salicylic acid (*β* = − 412) and apigenin (*β* = 722), respectively (Table 5). Therefore, morphological variables were involved in the synthesis and accumulation of these compounds. Khadivi-Khub et al.^57^, reported the relationship between morphological and phytochemical parameters. Research on the correlation of these traits can help plant breeders in selecting suitable ecotypes.

**Table 5:** Phytochemical compounds related with morphological traits using multiple regression analysis.

Traits	Morphological parameter	*r*	*R*^2^	Standardized beta coefficients	*t* value	*p* value
Palmitic acid	1000 seed weight	0.599	0.359	− 0.599	− 4.239	0.015
Vaccenic acid	Pericarp weight	0.642	0.412	0.642	− 3.213	0.008
Arachidonic acid	1000 seed weight	0.679	0.461	0.679	2.774	0.022
Salicylic acid	Pericarp weight	0.585	0.342	0.349	3.498	0.017
	Fruit length	0.809	0.453	− 0.412	− 4.345	0.044
Apigenin	Fruit weight	0.722	0.522	0.722	5.312	0.028
Total carotenoids	Pericarp weight	0.761	0.579	− 0.620	− 3.405	0.009
	1000 seed weight	0.872	0.761	0.449	2.468	0.039

### Canonical correspondence analysis

**Figure 9:**
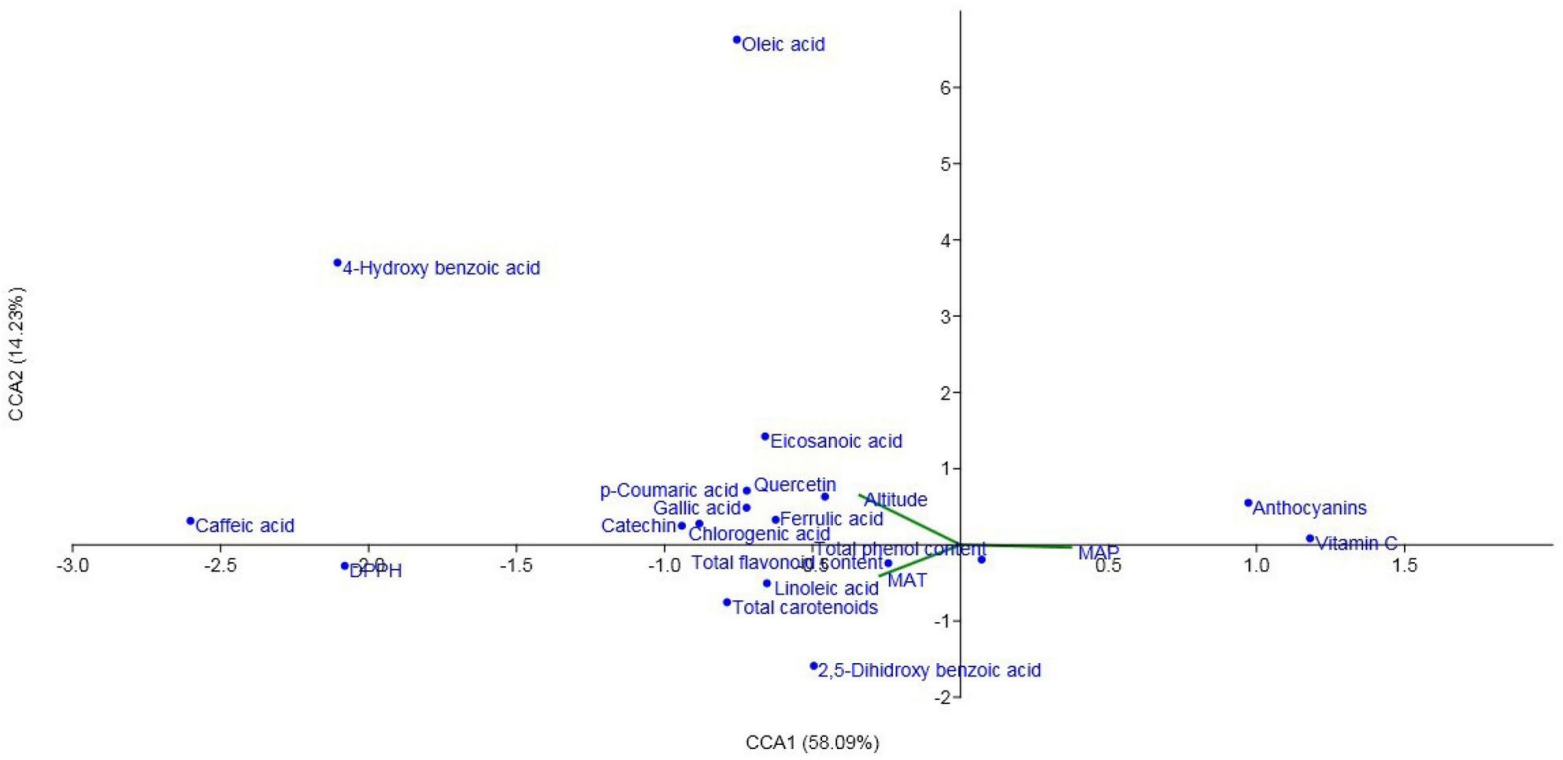
Canonical correspondence analysis biplot of *Rosa canina* ecotypes, linking percentages of the major and important constituents, collected from different environmental conditions. *MAT* mean annual temperature, *MAP* mean annual precipitation.

The CCA was performed to evaluate the correlation between the studied phytochemical compounds and three environmental factors such as mean annual precipitation (MAP), altitude, and mean annual temperature (MAT) (Fig. 9). The RCEs are distributed within the latitude of 30° 22ʹ N to 38° 16 ʹ N and longitude of 45° 34 ʹ E to 50° 55 ʹ E including different geographical regions. The mean rainfall of the RCEs is between 200 and 895 mm/year. The first CCA variable (CCA1) concerning environmental factors presented that MAP had a positive share, while MAT and altitude had a negative share on this CCA construction. The second CCA (CCA2) variable in connection to the phytochemical traits showed that the most of the compounds had a negative share in the formation of CCA variables. Vitamin C, anthocyanins content, and TPC had a positive share with MAP. Geographical conditions, genetic factors, and the different potency to synthesize are involved of specialized metabolite contents^56^.

## Conclusion

Rose hip is used as a raw material in the pharmaceutical, cosmetic, and food industries. Production of modified varieties with desirable agromorphological and phytochemical traits based on the needs of these industries seems to be essential. In the present study, a considerable diversity between the Iranian RCEs was observed in terms of morphological, and phytochemical traits such as fatty acid compounds. This study offered novel information on the fatty acid composition in rose hip seeds and pertinent oils derived, as well as the content of vitamin C, anthocyanins, carotenoid, and phenolic compounds content of the fruit pericarp from Iranian wild RCEs. The initial evaluation of RCEs in terms of morphological, and phytochemical traits can help to introduce suitable genotypes for cultivation and use in the pharmaceutical, food, and cosmetic industries, and also the best parents can be selected for the improvement of this plant and used in breeding programs.

## Data Availability

The datasets used during the current study available from the corresponding author on reasonable request.
